# FliL association with flagellar stator in the sodium-driven *Vibrio* motor characterized by the fluorescent microscopy

**DOI:** 10.1038/s41598-018-29447-x

**Published:** 2018-07-24

**Authors:** Tsai-Shun Lin, Shiwei Zhu, Seiji Kojima, Michio Homma, Chien-Jung Lo

**Affiliations:** 1 Department of Physics and Graduate Institute of Biophysics, National Central University, Jhongli, Taiwan 32001 Republic of China; 2 Division of Biological Science, Graduate School of Science, Nagoya University, Chikusa-ku, Nagoya 464-8602 Japan; 3 Department of Microbial Pathogenesis, Microbial Sciences Institute, Yale School of Medicine, New Haven, CT 06536 USA

## Abstract

Bacterial flagellar motor (BFM) is a protein complex used for bacterial motility and chemotaxis that involves in energy transformation, torque generation and switching. FliL is a single-transmembrane protein associated with flagellar motor function. We performed biochemical and biophysical approaches to investigate the functional roles of FliL associated with stator-units. Firstly, we found the periplasmic region of FliL is crucial for its polar localization. Also, the plug mutation in stator-unit affected the polar localization of FliL implying the activation of stator-unit is important for FliL recruitment. Secondly, we applied single-molecule fluorescent microscopy to study the role of FliL in stator-unit assembly. Using molecular counting by photobleaching, we found the stoichiometry of stator-unit and FliL protein would be 1:1 in a functional motor. Moreover, the turnover time of stator-units are slightly increased in the absence of FliL. By further investigation of protein dynamics on membrane, we found the diffusions of stator-units and FliL are independent. Surprisingly, the FliL diffusion rate without stator-units is unexpectedly slow indicating a protein-complex forming event. Our results suggest that FliL plays a supporting role to the stator in the BFM.

## Introduction

Motility is one of the most important capability for bacterial survival. Bacterial flagellum is a rotating motility organelle driving bacteria for chemotaxis. A flagellum is composed of a flagellar filament, a hook, and a basal body^1^. Flagellar torque is generated from the flagellar motor embedded in the cell envelope^2,3^. A functional bacterial flagellar motor (BFM) is composed of a rotor with several stator-units surrounded^4–7^. A rotor is formed by two ring-like structures: MS-ring and C-ring. MS-ring comprises about 26 copies of FliF. C-ring is made up of dozens of copies of FliG/FliM/FliN protein complex^8^ located beneath the MS-ring^9,10^. A rotor is responsible for the switching and torque generation via an interaction between the FliG and stator-units^11–13^. A stator-unit is a membrane protein complex with 4 MotA and 2 MotB stoichiometry^14,15^, functioning as two channels to conduct ions across the membrane and coupling to the torque generation^16^. There are up to a dozen stator-units in a functional motor when applying high load on a flagellar motor. A single stator-unit is capable of driving the rotor by conducting at least 37 ions/revolution^17^. Summaries of BFM functions and models can be found in several review reports^1,18,19^.

There are two major types of ions driving BFM: a H^+^-driven stator-unit complex is composed of MotA and MotB in *Escherichia coli* and *Salmonella enterica*; a Na^+^-driven stator-unit complex is composed of PomA and PomB in *Vibrio alginolyticus*^20,21^. However, both types of stator-units share several common features in the structure and function: (1) the A-subunit in the complex has 4 transmembrane segments. The B-subunit has a single transmembrane segment and a large periplasmic region containing a ‘plug’ segment and a peptidoglycan-binding domain required for the stator activation^22–24^. (2) Both types of stator-units are dynamic in response to the energetic and the mechanical load. A rapid turnover of MotAB stator-units has been revealed in the functioning motor in *E. coli*^6^. The same phenomenon of the assembly/disassembly of the PomAB, which is dependent on sodium ion influx, was revealed in *V. alginolyticus*^25^. Stator-units respond to the external load by regulating itself dynamic assembly around the rotor^26–30^. The stator-units may work as a mechanosensor in addition to an energy transferring conductor and a torque generating unit.

Unlike these well-known flagellar proteins, several reports addressed FliL’s role in the BFM function in different species with diverse phenotypes. The *fliL* mutant in *Caulobacter crescentus* had a paralyzed motility but FliL is not a part of flagellar basal body^31^. On the other hand, FliL is suggested to interact with the flagellar basal body as an inner membrane protein in *Salmonella*^32^. FliL defect has a mild effect on the swimming motility in *Salmonella*^33^ but causes an abolishment of swarming motility on agar plates resulting from massive filament release^34^. The swarming motility defect could be alleviated by the overexpression of FliL with the stator or increasing flagellar number^35,36^. FliL defect in *Proteus mirabilis* impairs both swimming and swarming motility due to having flagellar synthesis problem^37^. Sequence research on FliL in *P. mirabilis* suggested that FliL works as a surface sensor through regulating gene expression^38–40^. FliL defect in *Rhodobacter sphaeroides* results in an impaired swimming motility^41^. The orientation of periplasmic flagella in *Borrelia burgdorferi* is altered due to *fliL* gene deleted^42^.

Recently, there are two important finding regarding FliL’s role in BFM function. Firstly, a flagellar motor structure *in situ* resolved by cryo-electron tomography showed that a cytoplasmic membrane protein, FliL locates between the stator and rotor^42^. Secondly, the recent two papers found that FliL is involved in torque generation of the flagellar motor in high load environment^43,44^. However, FliL localization to the basal body is dependent on the presence of stator-units in *V. alginolyticus* but repelled by the stator-units in *Salmonella*^43^. Furthermore, the stator-unit localization around the basal body is also dependent on FliL. Thus, it is important to clarify the functional relationship between stator-units and FliL to obtain a comprehensive understanding of the BFM mechanism.

*V. alginolyticus* is a Gram-negative marine bacterium having a single sheathed polar flagellum driven by sodium-motive force and numerous lateral flagella driven by proton-motive force^3,18^. Each flagellum contains distinct FliL in their motor; the polar FliL and lateral FliL. The polar FliL has been studied well but the lateral FliL has not yet been characterized. The sodium-driven motor is a good candidate for investigating motor working mechanism^17^. In this report, we focused on the polar FliL and we found that FliL periplasmic region is important for its polar localization. And the plug region in stator-unit is necessary for recruitment of the FliL. We also used a *V. alginolyticus* mutant strain LPN4 with hyper sodium-driven flagella located at the lateral positions as an excellent model system to investigate the stator and FliL interaction^45^. We performed fluorescence recovery after photobleaching (FRAP) and single-molecules tracking on functioning flagellar motor to study stator abundance and dynamics with or without FliL. The stator-unit turnover rate is weakly affected by FliL and the dynamics on the membrane is unaffected without FliL. Surprisingly, the diffusion rate of FliL on the membrane is slow indicated an oligomer state formation on the membrane.

## Results

### Polar localization of FliL is mainly regulated by its periplasmic region not transmembrane region

**Figure 1:**
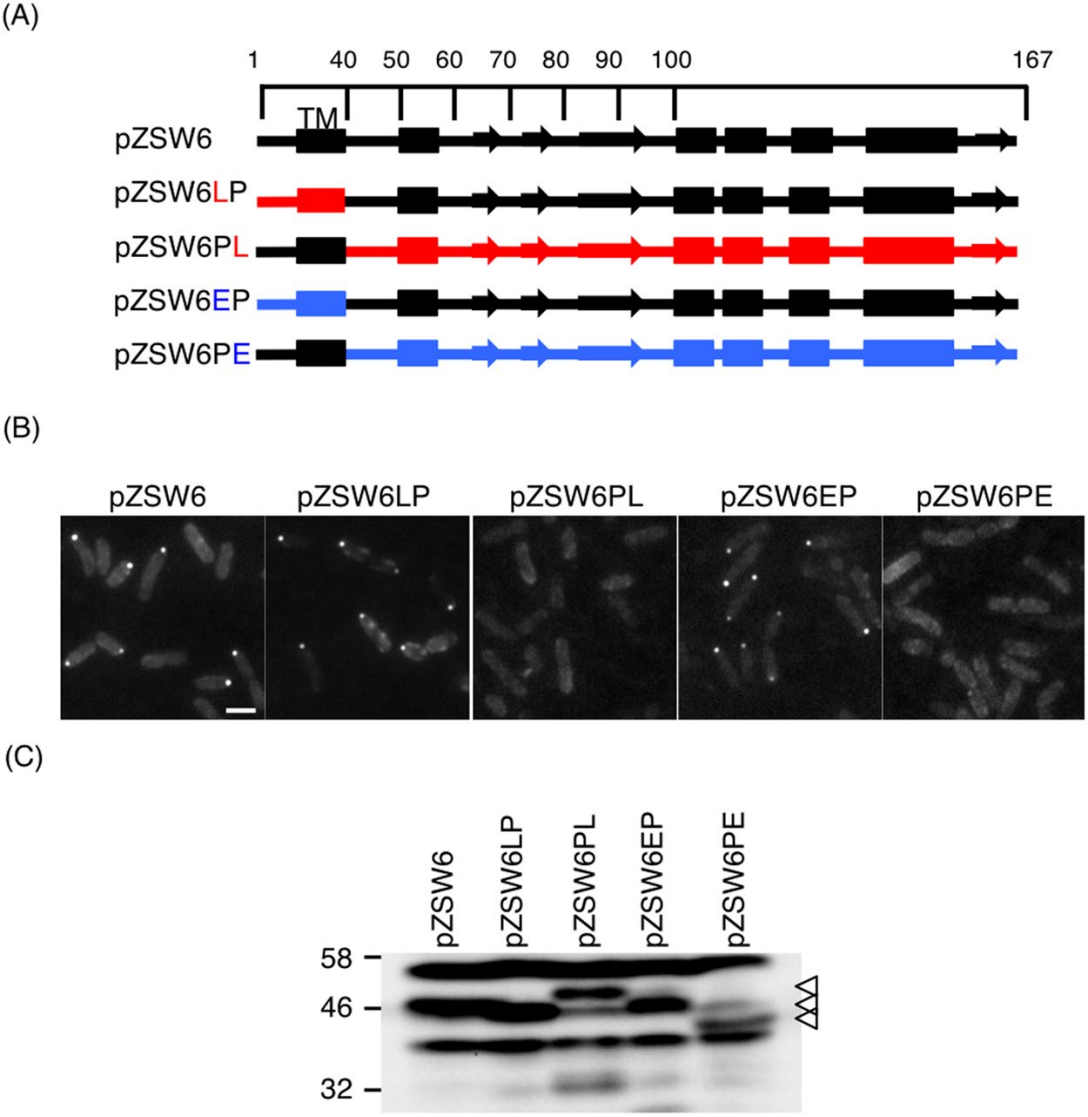
Periplasmic region of FliL is required for its polar localization. (**A**) A schematic of polar FliL or chimeric FliL. Protein second structure is predicted using Topcons^68^. The α-helix labeled in a rectangular shape. β-sheet in an arrow shape. N-terminal transmembrane segment of wild-type polar FliL ends at residues of L39. Black, red, and blue colors represent segments of polar FliL, lateral FliL of *V. alginolyticus* and *E. coli* FliL respectively. TM indicates the transmembrane region. (**B**) Representative fluorescent microscopic images of eGFP fused to polar FliL or chimeric FliL in ZSW1 cell. Scale bar = 2 μm. (**C**) Detection of chimeric FliL constructs fused with eGFP, by immunoblot using anti-eGFP antibody. White triangles indicate the positions of FliL fusion constructs. Full-length blot is shown in Fig. S1.

*V. alginolyticus* polar FliL is located in the base of polar flagellum^44^. Since FliL is a membrane protein, we made chimeric FliL constructs between polar FliL of *V. alginolyticus* and lateral FliL of *V. alginolyticus* or FliL of *E. coli* to characterize the key region for the polar localization of FliL. An eGFP-tag is fused to the N-terminus for FliL fluorescence observation^44^. Four chimeric FliL constructs were made based on pZSW6 as shown in Fig. 1A.

We introduced these plasmids into the ZSW1 strain that was generated by a deletion of polar *fliL* in the lateral flagella defective strain VIO5 (Table 1). The plasmid pZSW6LP and pZSW6EP, in which the polar FliL N-terminal region (residues, 1–39) was replaced with the corresponding region of *V. alginolyticus* lateral FliL and *E. coli* FliL, respectively, and fused to periplasmic region of *V. alginolyticus* polar FliL (a.a. 40–167), showing fluorescent dots in the pole of cell as pZSW6 which produces the wild-type polar FliL. On the contrary, when the large periplasmic domain of lateral FliL or *E. coli* FliL were fused to the C-terminal of polar FliL, the chimeric constructs did not a show fluorescent dot in the cell pole (Fig. 1B). The expression and stability of all constructs were analyzed by immunoblotting of whole-cell extracts. All constructs except the pZSW6PE showed a detectable band of fusion protein around 46 kDa (Figs 1C and S1), indicating that the chimeric FliL constructs fused with a eGFP-tag are relatively stable. Thus, the periplasmic region is required for its polar localization.

**Table 1:** Table of strains and plasmids.

Strain or plasmid	Genotype or description	Source
*V. alginolyticus*		
VIO5	VIK4 *laf (Rif* ^*r*^*Pof*^+^*Laf*^−^)	Okunish *et al*.^63^
LPN4	VIO5 Δ*flhFG* Δ*sflA*	Kitaoka *et al*.^45^
ZSW1	VIO5 Δ*polar fliL*	Zhu *et al*.^44^
ZSW2	VIO5 Δ*polar fliL* Δ*pomAB*	Zhu *et al*.^44^
ZSW5	LPN4 Δ*pomAB*	This study
ZSW6	LPN4 Δ*polar fliL* Δ*pomAB*	This study
*E. coli*		
DH5a	Recipient for cloning experiments	
β3914	β2163 gyrA462 zei-198::Tn10(*Km*^*r*^*Em*^*r*^*Tc*^*r*^)	Le Roux *et al*.^59^
Plasmids		
pSW7848	Suicide plasmid for allele exchange - oriVR6K γ oriTRP4	Val *et al*.^58^
pZSW2	pSW7848/flanking regions (500 bp) of *fliL*	Zhu *et al*.^44^
pKY704-Δ*pomAB*	pKY704-pomA1–68-pomB126–301, that was constructed by the *DraI* and *HpaI* deletion	Yorimitsu *et al*.^64^
pGEM-T	TA cloning vector	Promega
pBAD33	araBAD promoter, *Cm*^*r*^	Guzman *et al*.^65^
pHFAB	pBAD33/*pomA* + *pomB*	Fukuoka *et al*.^66^
pHFGBA2	pBAD33/*his6-egfp-pomB, pomA*	Fukuoka *et al*.^66^
pZSW6	pBAD33/*egfp-polar fliL*	Zhu *et al*.^44^
pZSW6ΔL	pBAD33/*egfp-polar fliL with in-frames deletion from residues 40 to 50*	This study
pZSW6LP	pBAD33/*egfp-chimeral pfliL with N-terminal replacement with lateral FliL*	This study
pZSW6PL	pBAD33/*egfp-chimeral pfliL with C-terminal replacement with lateral FliL*	This study
pZSW6EP	pBAD33/*egfp-chimeral pfliL with N-terminal replacement with E. coli FliL*	This study
pZSW6PE	pBAD33/*egfp-chimeral pfliL with C-terminal replacement with E. coli FliL*	This study
pZSW7	pBAD33/*pfliL-pomAB*	This study
pZSW81	pBAD33/*egfp-pfliL-pomAB*	This study
pZSW81-B1	pBAD33/*egfp-pfliL-pomAB* Δ*plug(*Δ*44*–*58 in pomB region)*	This study
pTY200	pBAD33/*egfp*	Yorimitsu *et al*.^67^

### The plug in the stator protein of PomB is important for FliL polar localization

**Figure 2:**
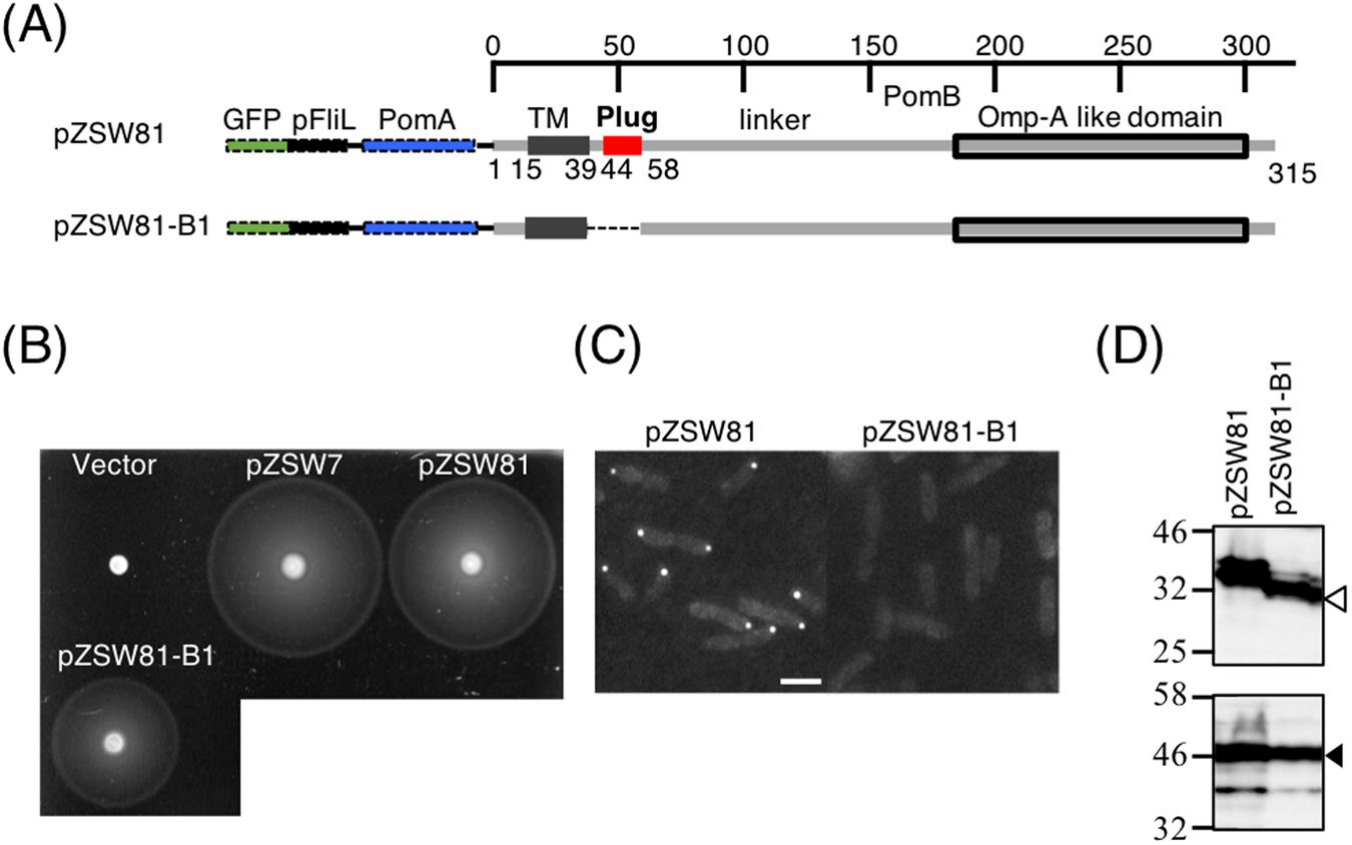
The plug in the periplasmic region of stator is crucial for FliL polar localization. (**A**) A scheme of the plasmids. eGFP, FliL and PomA constructs are labeled in dashed lines, and they do not reflect the real length. PomB is shown in the gray line and an in-frame deletion of the periplasmic plug region (a.a. 44–58) is shown in dash lines. (**B**) Motility assay of ZSW2 strains harboring different plasmids. Vector, pZSW7 encoding both *pomAB* and *fliL* and pZSW81 series of plasmids were introduced into ZSW2. Overnight cultures of each strain were spotted on the soft agar plate containing 0.02% arabinose and 2.5 μg/mL chloramphenicol and incubated for 6 hours at 30 °C. (**C**) Representative fluorescent microscopic images of ZSW2 cell expressed eGFP-pFliL in presence of stator (pZSW81) or of plug deleted stator (pZSW81-B1). Scale bar = 2 μm. (**D**) Protein detection of eGFP-FliL, stator and stator with a plug deletion. Protein detection from whole cell extracts by immunoblot using anti-PomBc antibody is indicated by a hollow triangle. Protein detection of eGFP-FliL by immunoblot using anti-FliLc antibody is indicated by a solid triangle. Full-length blot is shown in Fig. S2.

A previous report showed that in the absence of stator-units, the polar localization of FliL is not observed^44^. Because the periplasmic region of FliL is important for its polar localization, we put our focus on the periplasmic domain in the B subunit of stator. PomB has a large periplasmic domain that consists of a “plug”, a linker and an OmpA-like domain^46^. Because the linker and the OmpA-like domain are suggested for peptidoglycan binding during stator activation, we mainly investigate the role of “plug” region for stator and FliL interaction. We constructed a plasmid expressing eGFP-FliL, PomA and PomB from the arabinose inducible promoter and named it as pZSW81, and deleted the plug region of PomB to generate pZSW81-B1(Fig. 2A). The plasmid expressing polar FliL, PomA and PomB (pZSW7) was introduced to the *fliL and pomAB* deletion strain ZSW2, and this strain complemented the defect of ZSW2, showing the wild-type motility. All constructs were incubated on a soft agar plate to test swimming ability. The ZSW2 strain harboring pZSW81 showed a similar swimming ring size to that of pZSW7, indicating that the fusion with eGFP-tag to FliL does not affect both FliL and stator function (Fig. 2B). Although the “plug” deleted PomB mutant expressed from pZSW81-B1 has a moderate effect on swimming motility (Fig. 2B) by causing ion leakage through the stator into the cell^46,47^, an obvious swimming ring could be obtained indicating that “plug” deleted stator still worked (Fig. 2B). However, no fluorescent dot of FliL at the cell pole was visible in pZSW81-B1 (Fig. 2C), indicating that the localization as the part of the function is affected by the plug deletion. Since immunoblot analysis showed that both eGFP-FliL (lower panel) and PomB (upper panel) constructs expressed from pZSW81 and pZSW81-B1 were detected at similar levels without degradation (Figs 2D and S2), We exclude the possibility that the loss of polar localization in pZSW81-B1 was not caused by the protein instability. Thus, these results suggest that the plug region in the stator is crucial for polar localization of FliL.

### Stator abundance in both the presence of FliL and the defect of FliL

**Figure 3:**
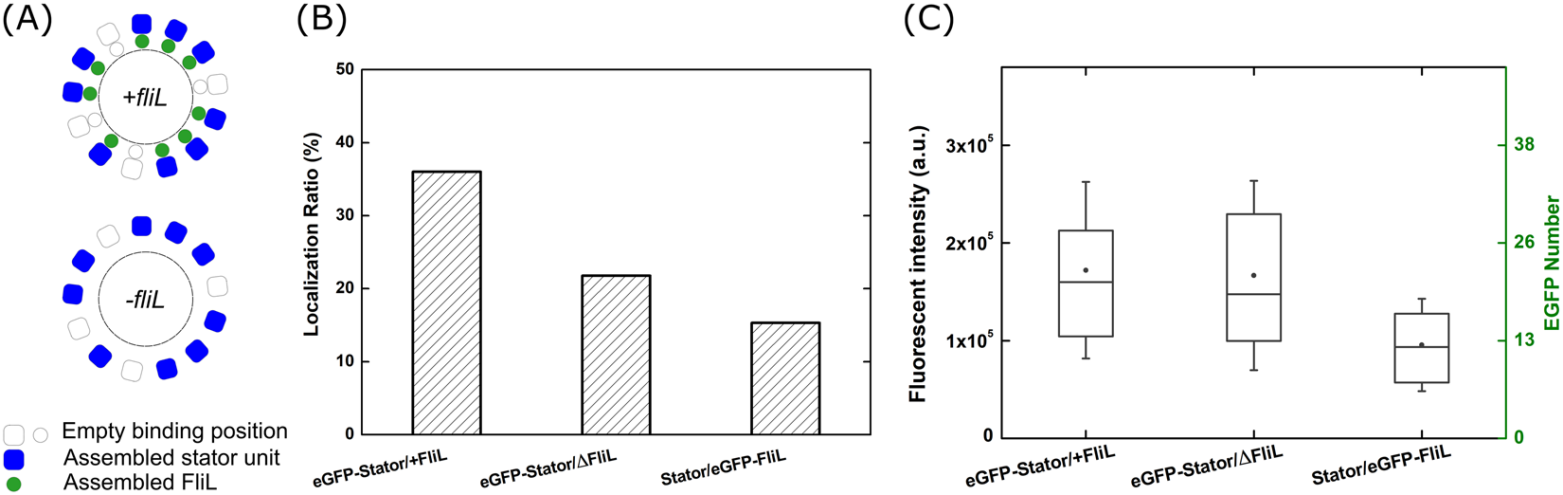
Stator-units and FliL stoichiometry. (**A**) Schematic of the experimental strain design. Stator-units assembled to the BFM with/without FliL. (**B**) Protein localization ratio on different strains. Localization ratio represents cells having foci spots in whole observation population. eGFP-Stator/+FliL (ZSW5/pHFGBA2) and eGFP-Stator/ΔFliL (ZSW6/pHFGBA2) are strains carrying eGFP fused to PomB with/without FliL expression respectively. Stators/eGFP-FliL (ZSW6/pZSW81) strain carries eGFP fused to FliL. Counted cell number from left to right: 2562, 1963, 1773. (**C**) Statistical results of stator-units and FliL numbers in BFM. The left vertical axis is the total fluorescent intensity (F_T_) in the motor region. Right vertical axis is the number of eGFP. The box indicates the interquartile range of the data with 25th percentile and 75th percentile at the edge. Medium is the line in the box. Filled circle is the mean of the data. The whisker indicates the standard deviation. Data number for each strain from left to right: 77, 29, 32.

From these biochemical results, the localization of polar FliL has high dependency on functional stator-units. And recent research suggesting FliL would affect the torque generation, which could couple with the stator-unit number around a motor^43,44^. We performed single molecular counting by photobleaching the stationary foci to obtain the abundance of assembled stator-units or FliL in a functioning motor (Fig. 3A)^6,48^. We used a mutant strain, LPN4, with polar flagella motors but laterally distributed that is suitable for the observation of stator abundance and turnover in TIRF microscopy. We deleted *pomAB* gene in the background of LPN4 to generate the strain ZSW5 (Table 1). We further deleted polar *fliL* gene in the strain of ZSW5 to generate the ZSW6 strain (Table 1). We focused on three strains, ZSW5/pHFGBA2 (eGFP-Stator/+FliL), ZSW6/pHFGBA2 (eGFP-Stator/ΔFliL) and ZSW6/pZSW81 (Stator/eGFP-FliL). All three strains are motile and rotating in tethered assay indicating the eGFP-fused proteins didn’t disturb BFM functions.

Under epi-fluorescent microscope, we counted the localization ratio for three strains, eGFP-stator with FliL, eGFP-stator without FliL, and eGFP-FliL with stator (Fig. 3B). Comparing the eGFP-stator with and without FliL strains, the ratio is similar to the previous results^44^ that decreased from 36% to 22% as the FliL defected. Surprisingly, the eGFP-FliL localization ratio is lower about 15% suggesting the FliL assembly affinity may be lower than stator-units.

To count the number of stator-unit proteins, we measured the fluorescent intensity of foci (Fig. 3C). The total fluorescent intensity (F_T-stator_) of the foci are the same in the presence of FliL (eGFP-Stator/+FliL) and the absence of FliL (eGFP-Stator/ΔFliL) indicating the number of stator-units are unaffected by FliL. We also calculate foci fluorescent intensity (F_T-FliL_) on eGFP-FliL (Stator/eGFP-FliL) strain. The average foci fluorescent intensity is about half of the stator-units foci, F_T-FliL_/F_T-stator_ = 0.56 ± 0.106 (standard error of the mean). Because an eGFP-stator has two eGFP-PomB, the result indicated the stoichiometry of stator-unit and FliL protein is close to 1:1. This result of *V. alginolyticus* is different from the two-hybrid analysis FliL-FliL interaction results measured in *E. coli* suggesting the ratio should be 1:2^43^.

Further, we performed the protein counting by photobleaching analysis that has been applied in the *E. coli* and *Shewanella*^6,48^ BFM studies. The single eGFP intensity (F_S_) can be found from photobleaching traces on the fluorescent foci and single particle traces (See Method and Fig. S3). The number of target proteins can be calculated by dividing the total eGFP fluorescent intensity to the single fluorophore fluorescent intensity, (N_p_ = F_T_/F_S_). We found about 22 PomB proteins in a BFM. There are 2 PomB subunits and 4 PomA subunits in one functional stator-unit^14,15^. In 0.006% arabinose induction case, about 11 ± 1 (standard error of the mean) stators are determined in the presence of FliL and in the deletion of FliL. For FliL protein, there are about 12 ± 1 FliL proteins in a motor (Fig. 3C). The number is close to the suggested full stator-units number (13 units) assembled around the rotor^49^ by EM image analysis. Therefore, we can conclude that FliL did not affect the stator-unit assembly and the stoichiometry of stator-unit and FliL is 1:1.

### Stator-units and FliL turnover dynamics

**Figure 4:**
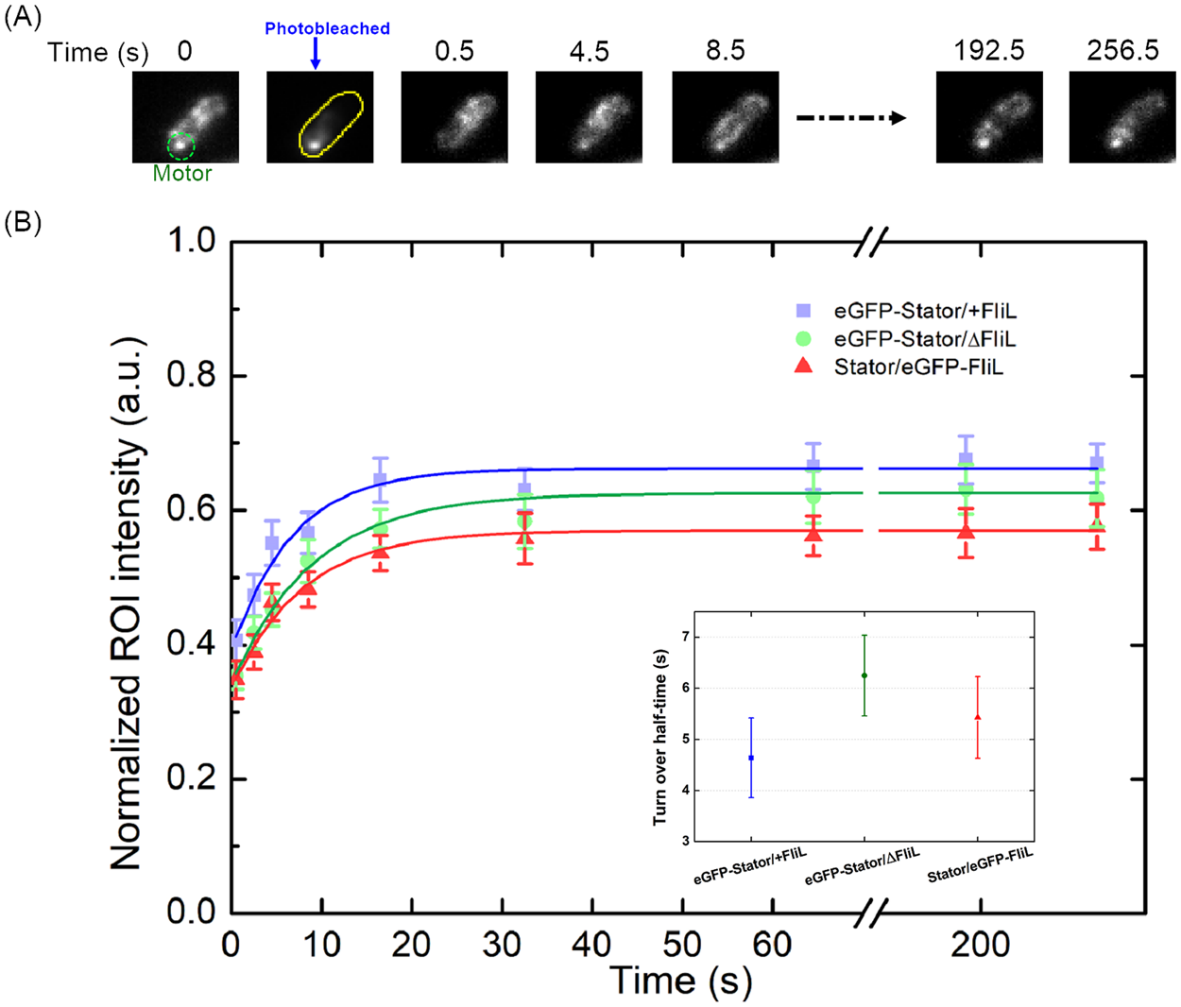
Stator-units and FliL dynamics on the flagellar motor. (**A**) Sample time-lapse images of fluorescent recovery after photobleaching experiments. A fluorescent spot of the assembled stator-units (green circle at time 0 s) was photobleached by a focus laser beam. The intensity of fluorescent spot region disappeared at time 0.5 (s) and then recovered by time. After about 8.5 s, there is a clear fluorescence spot in the same region. (**B**) The fluorescent recovery curves of three strains. Strains are the same as in Fig. 3, eGFP-Stator/+FliL (ZSW5/pHFGBA2), eGFP-Stator/ΔFliL (ZSW6/pHFGBA2), and stators/eGFP-FliL (ZSW6/pZSW81). Each color scatters with error bars represent the mean and standard error of the mean respectively from at least 25 cells. The curve was derived from fitting the theoretical recovery equation giving the turnover time^48^. The inset shows the turnover half-time (ln(2)/K_off_) with fitting errors for different strains.

To further investigate the role of FliL on the BFM stator dynamics, we performed fluorescence recovery after photobleaching (FRAP) on the eGFP-Stator or eGFP-FliL strains (Fig. 4A). We photobleached the fluorescent spot on motor and recorded the recovering process. Stator-units fluorescence recovery in the presence of FliL occurred at the rate of 0.149 s^−1^ (half-time ~ 4.64 s) and was moderately affected in the absence of FliL at the rate of 0.110 s^−1^ (half-time ~ 6.25 s) (Fig. 4B). The recovered fluorescence intensity of both strains are similar indicating the stator-units recruiting capability is not affected by FliL. On the other hand, FliL fluorescence recovery in the presence of stator-units occurred at the rate of 0.127 s^−1^ (half-time ~ 5.43 s) that is longer than the half-time of stator-units. Without stator-units, FliL is unable to localize to the rotor in *V. alginolyticus*. We speculate that FliL are recruited by stator-units and disengage from the motor after the stator-units absent.

Deducing from stator-units absent result in losing assembly of FliL on *V. alginolyticus*, the results suggested the FliL may disengage from motor after the stator-unit was absent that giving a slightly longer dissociating time in average. Since the recovery curves of eGFP-stator within or without the FliL shows slight difference, we suggested that the stator-unit turnover dynamic limitedly changed by the FliL.

### FliL does not affect the stator-units membrane diffusivity

**Figure 5:**
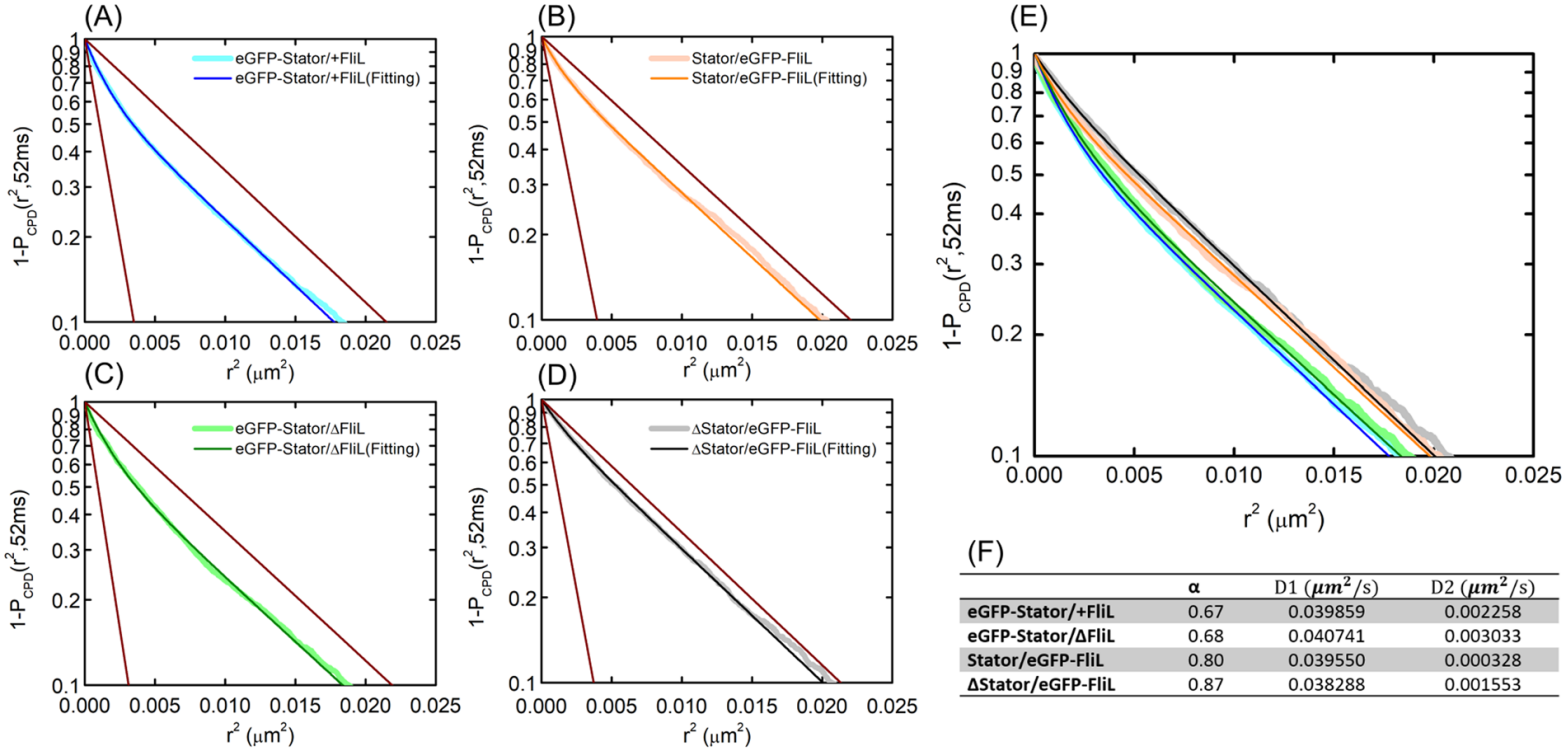
Protein dynamics. Cumulative probability distribution of single particle tracking. (**A**–**D**) The cumulative probability distribution (P_CPD_) at time interval 52 ms for different strains. The fade colored line was the experimental data. The deep color lines were the fitting solution from the experimental data. The brown lines represent the two diffusion coefficient components in CPD. (**E**) P_CPD_ data and fitting results plot together. The color symbols are the same as (**A**–**D**). (**F**) The table for the P_CPD_ fitting results of each strains.

The protein diffusivity on the membrane is one of the key parameters of membrane protein dynamics. We performed single molecules tracking of eGFP-Stator and eGFP-FliL on the membrane to obtain the quantitative diffusion constant. All of the fluorescent spots in the TIRF-image series were localized and tracked into position-time traces (See Methods). Typical protein position-time traces are too short to determine accurate diffusion constant using mean square displacement analysis. To obtain statistical reliable diffusion constant, we applied cumulative probability distribution (CPD) analysis in our experimental data (Fig. 5). CPD function describes the probability of a molecule to be found in a circle region from the origin at a given time lag. If a single molecule displacement follows Brownian motion, the CPD results to an exponential function^50–52^ (See Methods). There are two distinct states of stator-proteins and FliL, localized around the rotor and diffused on the membrane. Therefore, we use two components CPD functions to fit our data (see methods) that D1, D2, and α are the fast diffusion constant (membrane diffusion), slow diffusion constant (localization) and fast diffusion portion of the fitting data. We did cross-comparison for the diffusion of FliL and stator-unit on the membrane with/without the counterparts (Fig. 5F).

GFP fused stator-units with or without FliL (eGFP-Stator/+FliL, eGFP-Stator/ΔFliL) showed the same fast diffusion constant (0.04 μm^2^/s) with similar portion of free diffusion proteins (α = 0.68). The results showed that FliL does not affect the diffusivity of stator-units. We also measured the FliL dynamics with/without stator-unit (Stator/eGFP-FliL, ΔStator/eGFP-FliL). Surprisingly, the diffusion of FliL proteins is close to stator-unit that is about 0.04 μm^2^/s. For FliL only with a single transmembrane domain, the slow diffusion rate implied FliL might form a cluster itself. Without stator-units, most of the FliL proteins are diffusing and not localize in the BFM (α = 0.87).

## Discussion

Although *fliL* is a common gene in the bacteria that possess flagella, the main role of FliL remains unclear. Previous studies suggested FliL may involve in the torque-generation events^42–44^. FliL is mainly composed of a small N-terminal cytoplasmic region, single transmembrane segment, and C-terminal periplasmic region^44^. In this report, we applied biochemical and biophysical approaches to investigate the interaction between FliL and stator-units. Firstly, by using chimeric constructs of FliL, we found that the periplasmic region of FliL is required for its polar localization. Another possible explanation is the chimeric FliL with C-terminal replacement becomes less stable affecting the interaction to stator-units. It is notable that a recent report suggested that several components are strongly interacted with FliL including MotA in case of *Salmonella* flagellar motor^43^. However, such kind of interaction might be not mainly responsible for FliL in *V. alginolyticus*, since a replacement with lateral FliL and *E. coli* FliL in the N-terminus containing cytoplasmic region and transmembrane region does not affect FliL polar localization. We concluded that the periplasmic domain is required for the FliL function. Thus, the atomic structure of FliL is one of the key knowledge to understand the interaction between FliL and stator-units.

Secondly, we found that FliL is only recruited by stator-units which possess functional plug that is important in activation of stator-units^53^. The unplugged stator-units, which have assembling capability around the rotor, failed to recruit FliL (Fig. 2C). Similarly, although the FliL in *Rhodobacter sphaeroides* does not interact with periplasmic region of MotB^41^, the FliL could participate in the MotB coupling with BFM in an indirect fashion. Furthermore, motility defect caused by *fliL* deletion could be alleviated by mutations in the plug region of the stator in *E. coli*, also suggesting that the FliL might favor the unplug state of stator^43^. As suggested in many reports, when stator-units assemble around the rotor, FliG-PomA interaction will trigger the plug to be open and consequently to be unplugging state causing ion flowing through its complex^11,22,48,53,54^. Thus, we suggest that the plug of stator-units at open state might interact with the FliL periplasmic domain to allow stator in a more stable state.

Thirdly, we applied protein counting by photobleaching to determine the target protein abundance. In the presence of FliL, there are about 11 ± 1 stator-units in a motor that is consistent with a recent cryo-ET report suggest there are 13 stator-units^49^. The stator abundance experiment suggested that lacking FliL protein did not directly affect the final number for stator-units assembling. Recently published results by measuring tethering speed steps approaching showed a similar results^27^. The load condition for our experiments is at stall torque because the poly-l-lysine would tether the sheathed flagellum^55^. But it is surprising that the reducing in localization ratio did not consist with the stator-units number assembling on motor. One possible explanation is that FliL may support motor construction in the earlier stage to affect the localization ratio. For example, the FliL deleted strain in *Borrelia burgdorferi* would lead to altered periplasmic flagellar orientation^42^. In addition, we found that the stoichiometry of the stator and the FliL in *V. alginolyticus* flagellar motor could be 1:1 (Fig. 3C). However, it is worthy to note that a previous report suggested the stoichiometry of the stator and the FliL in the *Salmonella* to be 1:2^43^. They performed two-hybrid assay in the cytoplasmic of bacterial cell and pull-down assay, detected FliL-FliL interaction and thus suggested that a dimer of the FliL might exist in the flagellar motor. It is possible that FliL-FliL interaction is on the membrane but not inside the motor system. Considering the localization ratio in FliL is lower than stator-unit, it is possible to underestimate the number. However, the localization ratio maybe not directly represent the number of proteins assembly because the FliL defected did not affect final the number of stator-units on the motor. Further investigation is needed to clarify if there is a universal role of FliL in different bacteria.

Fourth, we applied FRAP experiments to reveal the stator-units and FliL turnover behavior between motor region and membrane pool. The stator-units turnover half-time in the presence of FliL is 4.64 ± 0.78 seconds and slightly increased to 6.25 ± 0.79 seconds in the absence of the FliL. The results suggested that FliL increases the dynamics of stator-unit. It implied FliL could speed up the stator-unit adaption which may contribute to the torque export. Surprisingly, FliL also dynamically turnover around the motor with the half-time of 5.43 ± 0.80 that is slightly slower than stator-units. This results suggested the FliL may disengage from the rotor after stator-unit left. Thus, FliL may be as a follower to support stator-units for adapting the spacing.

Finally, we applied a direct single molecular tracking to measure the dynamics of FliL and stator-units. We found the stator-units diffusion rate is not affected by the FliL. Compare to previous measurement of stator-units on *E. coli* membrane^6^, the diffusion constant of MotAB stator-unit is slower (0.008 μm^2^/s) than PomAB stator-units in *V. alginolyticus* (0.04 μm^2^/s). We speculate the cell envelop properties are different in these strains. In both FRAP and single-molecular tracking experiments, we found the stator-units dynamics are faster in *V. alginolytics*. Further, by the cross-comparison of FliL and stator-units diffusivity, we conclude that the FliL and stator-units are independent on the membrane. Since the protein diffusion on membrane usually dominated by the number of transmembrane segment^56^, it’s unusual to measure such a low diffusion rate for a single transmembrane FliL protein. According to the recent biochemical characterization^57^, FliL without transmembrane domain would interact with each other to form oligomer. These results highly suggest that FliL protein may diffuse on the membrane as an oligomer.

**Figure 6:**
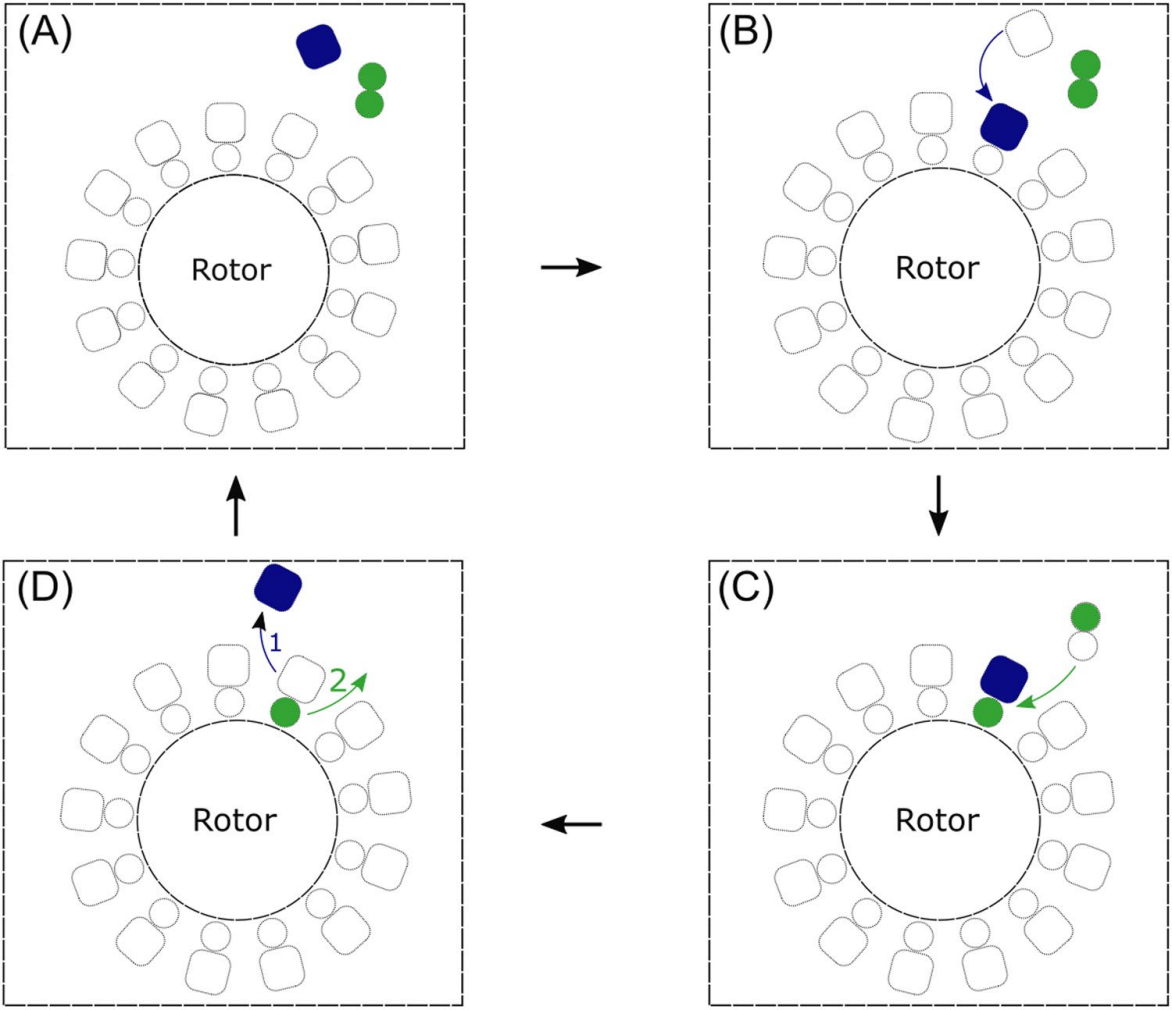
Model diagram of the suggesting association of FliL and stator-units. (**A**) The central circle is a rotor of a BFM. The blue square is a stator-unit. And the green twin circle represents FliL. They diffuse independently in the membrane pool. The circle and square with the dashed line around the rotor represent the possible position for FliL or stator-unit binding. (**B**) A stator-unit close to the rotor and activated by interacting with BFM. (**C**) The plug of stator-unit activation enables rotor to recruit a FliL. A FliL divided from the complex and bind to the rotor. (**D**) While a stator-unit leaves the rotor, the FliL would lose its stability and disengage from the rotor after the stator-units.

According to our results, we hypothesize a working model for FliL and stator-units interactions (Fig. 6). We suggest that FliL would form oligomer diffusing on the membrane and only recruit to the motor when stator-unit is activated on the motor. Somehow the FliL would change to single FliL to interact with stator-unit on the motor as 1:1 stoichiometry. And FliL would enhance stator-unit exchange rate. Once the stator-unit leaves the motor to be inactive, the FliL following to leave. In this model, FliL is in a supportive role of stator-units and torque generating. Further investigation is required to find out the critical role of FliL with other flagellar proteins.

In summary, we performed biochemical and biophysical study to directly observe the stator abundance with and without FliL. We revealed a novel role of the stator in *V. alginolyticus* species recruiting FliL through its plug region. We found that the stoichiometry of assembled stator and assembled FliL is 1:1. The turnover rate and the diffusivity of stator-unit don’t affect by the presence of FliL for the bacterial flagellar motor at the high load region. We speculate FliL has indirect or supporting role of stator-unit torque generation functions. Further Cryo-ET analysis and structure information is required for determining the accurate location of FliL and the true function in bacterial flagellar motor.

## Methods

### Strains, plasmids and growth conditions

Bacterial strains and plasmids used in this study are listed in Table 1. Regarding the deletion mutant of ZSW5 (*pomAB*) and ZSW6 (*pomAB* and *fliL*), 500 bp sequence upstream of the *fliL* or *pomA* start codon flanking with a *SacI* site at its 5′ end and 500 bp sequence downstream of the *fliL* or *pomB* stop codon flanking a *SacI* site at 3′ end were PCR amplified and these DNA fragments were together ligated into a pGEM-T cloning vector (Promega). After *SacI* digestion of this plasmid, the Δ*fliL* or Δ*pomAB* DNA fragment containing about 1000 bp was inserted into the suicide vector pSW7848^58^. Deletion strain ZSW5 and ZSW6 were constructed by using these suicide plasmids according to the published protocol^59^. The plasmids of pZSW7 and pZSW8 were generated using a fast-cloning method^60^. The constructed plasmids were introduced into *E. coli* DH5α, and cultured overnight at 37 °C in LB medium (1% Bacto tryptone, 0.5% yeast extract, 0.5% NaCl) containing 25 μg/ml chloramphenicol for plasmid collection. Plasmids were isolated by QIAprep Spin Miniprep Kit (Qiagen) and introduced into the *Vibrio* cell by electroporation with parameters of 1.4 kV and 200 W^61^. The sequences of all the plasmid constructs were checked using ABI Prism 3130 genetic analyzer (Applied Biosystems).

### Motility Assay

For the swimming assay on the soft agar plate, *V. alginolyticus* strains harboring vectors or plasmids (pHFAB or pZSW7) were cultured overnight at 30 °C on VC medium [0.5% polypeptone, 0.5% yeast extract, 3% (wt/vol) NaCl, 0.4% K_2_HPO_4_, 0.2% glucose] including 2.5 μg/mL chloramphenicol. Two microliters of overnight cultures were inoculated on the surface of VPG soft agar plates [1% bacto tryptone, 3% (wt/vol) NaCl, 0.4% K_2_HPO_4_, 0.5% glycerol, 0.25% bacto agar] including 2.5 μg/mL chloramphenicol and 0.006% or 0.02% arabinose.

For the swimming assay in tethered cell, we collected 200 ml cell culture after grown 4 hours in VPG broth. The cell pellets were collected and washed 3 times by TMN medium [50 mM Tris-HCl pH7.5, 5 mM glucose, 5 mM MgCl_2_, 500 mM NaCl]. The cell suspensions were passed through 2 syringes equipped with 26 gauge needles (TERUMO Hypodermic Needles: NN-2613R) for 50 times. Sheared cells were washed one time with TMN medium and incubated for 20 minutes at room temperature on a slide glass which has been coated with 200 times diluted anti-polar flagellin antibody. The tethered cells were observed by dark-field microscopy.

### Subcellular localization of FliL and stator and visualization flagella

Localization of the eGFP-fused FliL was observed as described previously^44,62^. In brief, ZSW1 cells harboring the plasmid a series of pZSW6 or ZSW2 cells introduced with a series of pZSW8 were cultured overnight in VC medium, then the culture were diluted 100 fold in VPG medium containing 0.006% arabinose inducer and chloramphenicol with 2.5 μg/mL final concentration. Cells were cultured for 4 h at 30 °C, then were harvested and resuspended in TMN medium. The resuspended cells were fixed with 0.1% (wt/vol) Poly-L-lysine and were observed by fluorescence microscopy (Olympus, BX50). The exposure time in this study for phase contrast is 0.01 second and for the fluorescence image is 1 second. For the observation of flagella, cells were incubated in TMN medium containing the anti-polar flagellum antibody for 5 minutes and were then washed twice in TMN medium. Cells were then incubated in TMN buffer containing an anti-rabbit IgG conjugated with rhodamine for 5 minutes. After washing the cells twice with TMN medium, the immunolabeled cells were observed using a fluorescence microscope.

### Detection of Proteins from Whole-Cell Lysate

Cells were cultured at 30 °C until they reached log-phase (typically 4 hours) in a VPG medium containing 0.02% arabinose and 2.5 μg/mL chloramphenicol. Cells were harvested and suspended in the SDS/PAGE loading buffer [66 mM Tris-HCl pH6.8, 8.3% (wt/vol) glycerol, 1% SDS, 16% (vol/vol) β-mercaptoethanol, 0.003% Bromophenol blue] at a cell concentration equivalent to an OD 660 of 10. After boiling the mixture at 95 °C for 5 minutes, 10 μl proteins mixture were loaded and separated by SDS-PAGE. Anti-eGFP antibody, anti-FliLc antibody or anti-PomBc antibody were used to detect GFP, FliL, and PomB proteins in the whole cell extracts by immunoblot.

### Localization ratio counting

Cells were cultured overnight in VC medium at 30 °C. Then the cells dilute 30 fold into VPG broth containing 2.5 μg/mL chloramphenicol and 0.006% arabinose as second culture at 30 °C for 4 hours to late-log phase. Then the cells were harvested and washed with TMN medium containing 100 μg/mL kanamycin twice by centrifuge. The cells were then loaded into a microscope chamber slide with pre-coated poly-L-lysine and 200 nm gold beads for 5 minutes. Unsettled cells were flushed out by TMN buffer. Then the slide was observed under a commercial microscope (Nikon Eclipse Ti-E) which is equipped with Zyla 4.2 Plus sCMOS camera, Omicro LedHUB^®^, and 100x Oil objective (Nikon, Apo TIRF 100x/1.49). The fluorescent image captured with LED 470 nm excitation for 1 second. The images were analyzed manually by ImageJ to count cells number and those cells have the fluorescent spot.

### Fluorescent abundance and protein membrane diffusivity

The cell preparation is the same as localization ratio counting. The slide was observed under a custom-build TIRF microscope with a 100x Oil objective (Nikon, Apo TIRF 100x/1.49), a 488 nm laser (Coherent, OBIS) for eGFP excitation, and an EMCCD (Photometrics, Evolve 512) for imaging recording. The details of abundance experiments can be found in the reference^6,48^. In short, TIRF photo-bleaching videos were recorded continuously with exposure time 50 ms for 600 frames. For the fluorescent abundance, we use the custom-wrote IDL to find a stationary spot in images. The fluorescent intensity bleaching over time was integrated by a region 320 ∗ 320 nm^2^ (5 pixels ∗ 5 pixels) for the first 200 frames. Then, the continuous photobleaching intensity traces were processed with Chung-Kennedy filter to preserve steps due to one eGFP photobleaching. The pairwise differences distribution function (PDDF) were calculated. Then we calculated the power spectrum from histogram of PDDF by FFT. By setting a high threshold, the highest frequency was extracted and inversed as single eGFP fluorescent intensity (Fig. S3). Because the data is usually noise, we also derived eGFP fluorescent intensity from the fluorescent spots diffusing on the membrane. By collecting the spot intensity, we can infer single eGFP fluorescent intensity as well.

For single-molecule tracking, we used custom-wrote IDL tracking program to calculate the molecule traces and analyzed the diffusivity of the proteins from previous continuous images. Each traces possess at least 5 points was used to calculate the cumulative probability distribution function (CPD), which statics the cumulative displacement probability at certain time-lag. Here we used 52 ms. We suppose the diffusion of proteins compose two kinds of normal diffusion. The cumulative probability distribution of two Brownian diffusion combination would be as the following equation,

1 $$1-{P}_{CPD}({r}^{2},\tau )=(\alpha )(ex{p}^{-\frac{{r}^{2}}{4{D}_{1}\tau +4{\sigma }^{2}}})+(1-\alpha )(ex{p}^{-\frac{{r}^{2}}{4{D}_{2}\tau +4{\sigma }^{2}}})$$

where P_CPD_ is the cumulative probability distribution of two Brownian diffusion rates. τ is the time lag at a given time. r is the displacement of particle from origin at a given time lag. D1, D2 are the diffusion coefficients. And σ is the localization errors. If the molecule exists two different rate Brownian motion. The probability to find a molecule in a circle region within radius r would be the sum of the two CPD with a certain ratio α between 0 to 1. The diffusion coefficients and ratio can be found by fitting our data to the equation (1).

### FRAP experiment

In FRAP experiment, cell preparation and setup are the same as described in doing fluorescent abundance. First, 3 frames were recorded to find a stationary spot. Then, the fluorescent spot was exposed under a centered focused laser spot for 0.5 s. After the fluorescent spot was photobleached, image series were recorded under TIRF for over 320 s with different time interval. The analysis processes referred from previously described^48^. The average intensity of fluorescent spot was normalized over each frame to eliminate the photobleaching effects by comparing the fluorescent intensity of the cell region (Equation 2). Before analysis, the image shifting was calibrated by measuring the gold beads position. Then, we used Gaussian fit to find the center of fluorescent spot. And the motor fluorescent intensity (***M***_*roi*_) was derived as mentioned in fluorescent abundance. The cell intensity (Cell) was derived by integrating the intensity in cell region, which was segmented by Niblack’s local threshold method at the first frame. And the background (B) is randomly selected dark region, which size is the same as ***M***_*roi*_ outside of the cell. Due to the experimental noise, all the data were averaged to get the recovery curve. The turnover rate of the stator was calculated by fitting exponential to the average curve by Origin 8.

2 $${I}_{N}(t)=\frac{{M}_{roi}(t)-B(t)}{{M}_{roi}(0)-B(0)}\cdot \frac{Cell(0)-B(0)}{Cell(t)-B(t)}$$

## Electronic supplementary material

Supplementary Information
