# Supplementary material for: FliL association with flagellar stator in the sodium-driven Vibrio motor characterized by the fluorescent microscopy
Source: Sci Rep. 2018 Jul 24;8:11172. doi: 10.1038/s41598-018-29447-x (PMC6057877; doi:10.1038/s41598-018-29447-x)
Supplement: Supplementary file 1 — Supplementary Information [file 41598_2018_29447_MOESM1_ESM.pdf]

Supplementary information

FliL association with flagella stator in the sodium-driven *Vibrio* motor characterized by fluorescent microscopy

Tsai-Shun Lin, Shiwei Zhu, Seiji Kojima, Michio Homma and Chien-Jung Lo

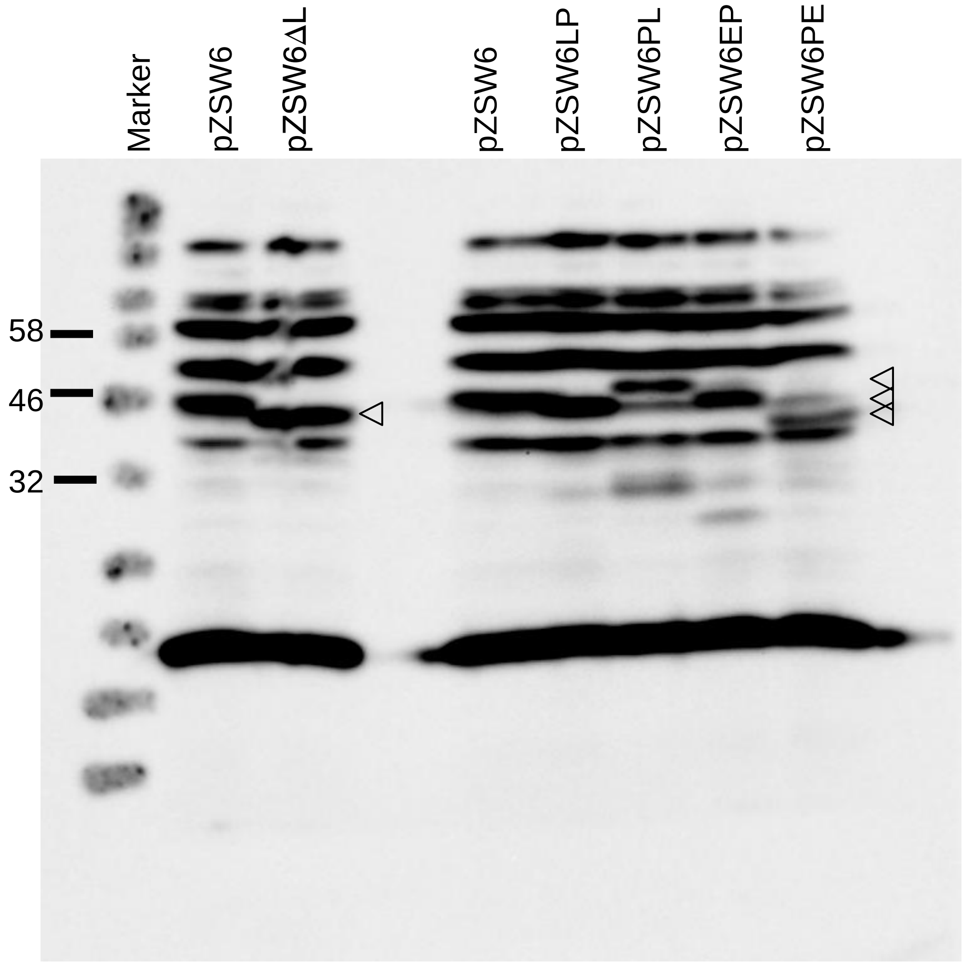

Fig. S1. Protein expression level detected by western blot analysis of a full-length blot is shown. A part of the blot data is shown in Fig. 1C. Marker: molecular weight marker (unit: kDa). Because monoclonal antibody against eGFP (BD living colors, Clontech) used in the current study reacted to other than eGFP, a lot of non-specific bands are visible in a full length of original blot. To confirm which band is representing our targeting protein, we conducted an in-frame deletion (residues from 40 to 50) at the *fliL* to detect the difference. Thus, the expression band shown in a triangle symbol represents the protein of eGFP-FliL.

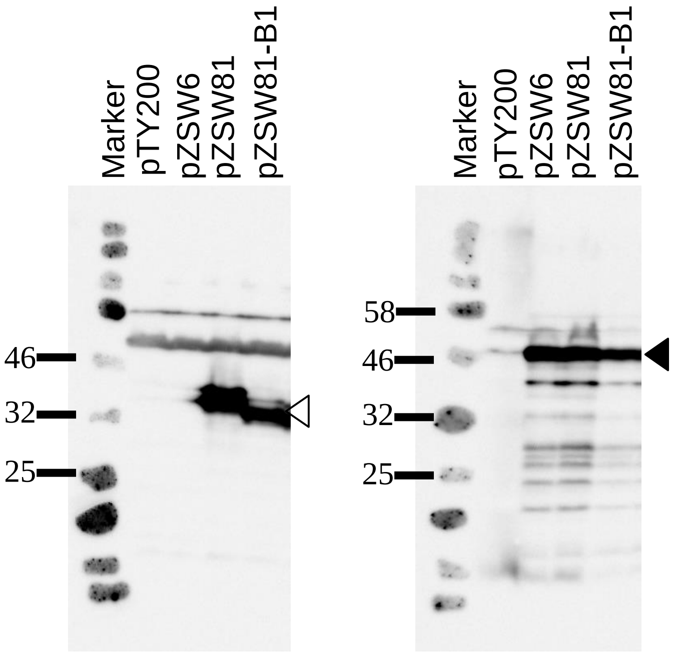

Fig. S2. Protein expression level detected by western blot analysis of full-length blots are shown. A part of the blots are shown in Fig. 2D. The expression band labeled in a white triangle is representing the stator B subunit; the protein of eGFP-FliL in a black triangle. Marker, molecular weight marker (unit: kDa).

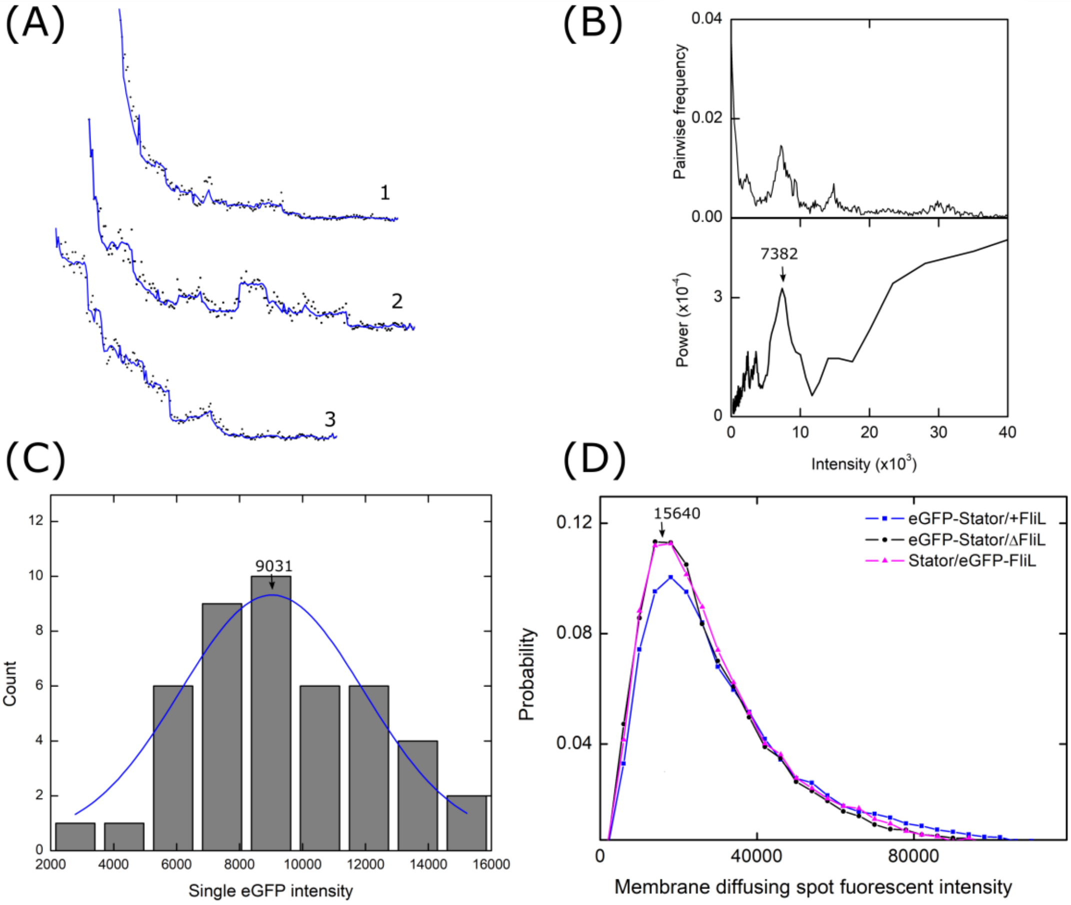

Fig. S3. Single eGFP fluorescent intensity analysis. (A) Three photobleaching traces from different strains for instance. Traces 1 to 3 were derived from eGFP-Stator/+FliL, eGFP-Stator/ΔFliL, and Stator/eGFP-FliL respectively. (B) Data processing from trace 1. The top figure shows the PDDF of the trace. And the Bottom figure revealed the power spectrum of the PDDF. The result shows a peak at intensity 7382. (C) Estimated single eGFP fluorescent intensity from histogram of photobleaching traces in all three strains data (45 traces). The blue line is a Gaussian fitting with a center at 9031. (D) Estimated single eGFP intensity from membrane eGFP spot tracking. The figure shows the fluorescent intensity distribution of the single molecular tracking from different strains. The peak 15640 is used to decide single eGFP fluorescent intensity. Since eGFP fusion on PomB, we speculate there should have two eGFP contributions. The peak was divided by 2 to get single eGFP fluorescent intensity about 7820. Since the values are similar from two methods, we set the 7820 as single eGFP fluorescent because the data number is much more than photobleaching traces.
